# Hyphae of the fungus *Aspergillus nidulans* demonstrate chemotropism to nutrients and pH

**DOI:** 10.1371/journal.pbio.3002726

**Published:** 2024-07-30

**Authors:** Riho Yamamoto, Hinata Miki, Ayaka Itani, Norio Takeshita

**Affiliations:** Microbiology Research Center for Sustainability (MiCS), Faculty of Life and Environmental Sciences, University of Tsukuba, Tsukuba, Japan; Duke University Medical Center, UNITED STATES OF AMERICA

## Abstract

The importance of fungi in ecological systems and pathogenicity hinges on their ability to search for nutrients, substrates, and hosts. Despite this, the question of whether fungal hyphae exhibit chemotropism toward them remains largely unresolved and requires close examination at the cellular level. Here, we designed a microfluidic device to assess hyphal chemotropism of *Aspergillus nidulans* in response to carbon and nitrogen sources, as well as pH. Within this device, hyphae could determine their growth direction in a two-layer flow with distinct compositions that were adjacent but non-mixing. Under conditions with and without a carbon source, hyphae changed growth direction to remain in the presence of a carbon source, but it was still difficult to distinguish between differences in growth and chemotropism. Although nitrogen sources such as ammonia and nitrate are important for growth, the hyphae indicated negative chemotropism to avoid them depending on the specific transporters. This fungus grows equally well at the colony level in the pH range of 4 to 9, but the hyphae exhibited chemotropism to acidic pH. The proton pump PmaA is vital for the chemotropism to acid pH, while the master regulatory for pH adaptation PacC is not involved, suggesting that chemotropism and adaptive growth via gene expression regulation are distinct regulatory mechanisms. Despite various plasma membrane transporters are distributed across membranes except at the hyphal tip, the control of growth direction occurs at the tip. Finally, we explored the mechanisms linking these two phenomena, tip growth and chemotropism.

## Abstract 2

Fungi depend on their nutrient-seeking behavior for survival, yet how they find nutrients is not well understood. Using a microfluidic device, this study shows that hyphae of the fungus Aspergillus nidulans exhibit chemotropism towards nutrients, adjusting growth direction towards carbon sources and away from certain nitrogen sources and acidic pH.

## Introduction

Tropism refers to the directional growth or movement of an organism or a part of an organism in response to a particular stimulus, such as light, gravity, touch, or chemicals. Tropism is a fundamental process in the behavior of many living organisms, and it allows them to adapt and respond to changes in their environment. For example, the giant sporangiophores (fruiting body) of the fungus *Phycomyces* bend toward blue light (phototropism) via blue-light receptor [1,2] and display negative gravitropism [3]. Thigmotropism (contact) and galvanotropism (electrical currents) have also been observed in some fungi [4].

Microorganisms in the environment adapt to their changing surroundings by moving toward or away from beneficial or toxic chemicals. Bacterial chemotaxis is the movement of bacteria towards or away from a particular chemical stimulus in the environment [5]. Fungal chemotropism has been analyzed well regarding sexual and vegetative hyphal fusion with mating pheromones [6,7]; nevertheless, there is limited knowledge regarding chemotropism to nutrients or hosts. Most fungi grow by extending their hyphae and branching, which make up the mycelial network [8]. Fungal hyphae grow by apical extension, where the tips of the hyphae secrete enzymes that break down surrounding organic matter, allowing the fungus to absorb nutrients [8–10]. Fungi engage in interactions and perform functions with a diverse array of targets, encompassing human and plant pathogens, decomposers of plant biomass, and symbiotic partners with plant roots [11–13].

When fungal colonies alter their shape in response to beneficial or harmful chemicals, distinguishing whether the growth at that site was affected or if the hyphae exhibited chemotropism becomes challenging. This underscores the importance of analyzing hyphal chemotropism at the single-cell level for accurate monitoring. Within the phytopathogenic fungus *Fusarium oxysporum*, microconidia extend germtubes in the direction of gradients involving diverse nitrogen and carbon sources on agar medium, such as glutamate, aspartate, and glucose [7,14]. A comparable assessment in the fungal parasitic mold *Trichoderma atroviride* reveals a chemotropism in germtube elongation toward various nitrogen and carbon sources [15]. Moreover, *F*. *oxysporum* demonstrates the ability to discern the presence of host plants by detecting peroxidase released from the host roots, guiding spore germination towards the host [16,17]. In these experiments, the microscopic observation of spore germination direction is conducted. Nevertheless, the mechanism by which hyphae regulate the growth direction is still largely unknown.

Microfluidic devices provide precise regulation of environmental conditions within purposely designed spaces and, when integrated with live imaging, unveil novel aspects of mycelial growth [18–20]. In this study, we have established an assay system using microfluidic control technology to determine the chemotropism of hyphae in response to nutrients and pH at the cellular level. Within this device, hyphae exhibit the capability to choose their growth direction in a two-layer flow with distinct compositions, positioned adjacent to each other but remaining unmixed.

## Results

### Microfluidic device to monitor hyphal growth with and without glucose

**Fig 1:**
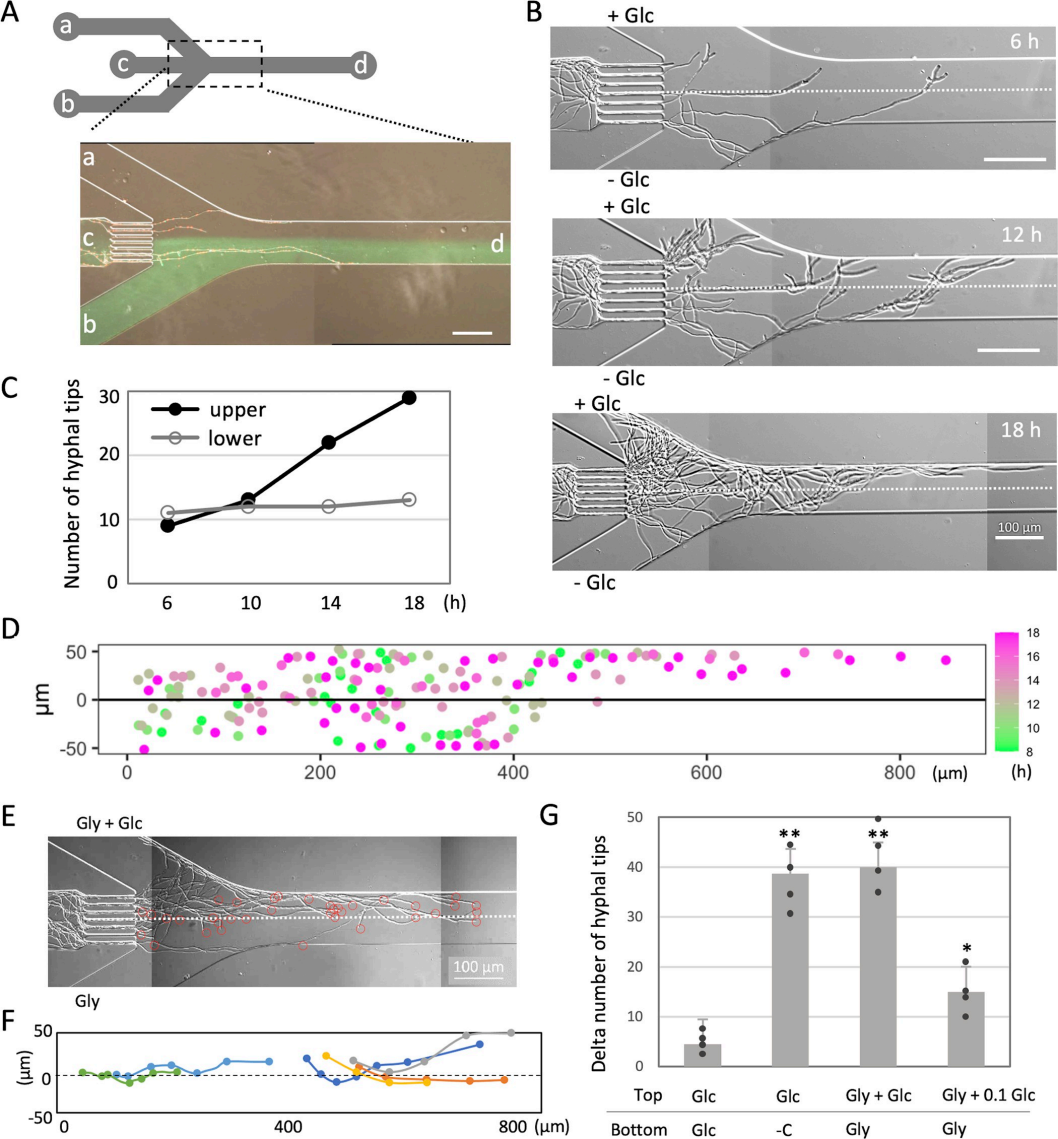
Microfluidic device to monitor hyphal growth in the presence and absence of glucose. (A) Y-shaped microfluidic device design. Spores were inoculated from the inlet (c) and trapped in 5-μm width channels. Hyphae grow towards the outlet (d) and are free to grow within the 2 layers merged from the inlet (a) and from the inlet (b). The media from (b) contain green-fluorescent dye. Scale bar: 100 μm. (B) Image sequence of hyphal growth in the condition with 1% glucose (upper) and no carbon source (lower) from S1 Movie. The elapsed time is given in hours. Scale bar: 100 μm. The border of 2 layers is indicated by the dot line. (C) Line graph of time variation in numbers of hyphal tips in the upper layer and the lower layer. (D) Merged plots of the position of the hyphal tips in the channel at each time in different color over time. (E) Hyphal growth in the condition with the upper layer containing 1% glycerol and 1% glucose and the lower layer containing 1% glycerol from S2 Movie. The positions of each hyphal tip are marked with red circles. The border of 2 layers is indicated by the dot line. Scale bars: 100 μm. (F) Tracking of directional change of hyphal tips around the border of 2 layers. Each trajectory is indicated by a different color. (G) The percentage difference in the number of hyphal tips in the upper and lower layers. Error bars represent standard deviations (SDs). *n* = 4; 40~50 hyphae in 3 independent experiments. **, *p* ≤ 0.01; *, *p* ≤ 0.05; ns, not significant. The data underlying this figure can be found in S1 Data.

Y-shaped microfluidic devices were fabricated, featuring a channel width of 100 μm (Fig 1A, see Materials and methods). These devices comprised 3 culture inlets (a-c) and 1 outlet (d). The 2 primary culture inlets (a, b) facilitated the controlled flow of 2 distinct media at appropriate rates, generating 2 adjacent layers where the media converged. Spores of the model fungus *Aspergillus nidulans* were introduced through the other inlet (c), captured in 5-μm channels, and allowed to undergo germination and growth while the liquid medium traversed through them. As the hyphae progressed through the device, they had the flexibility to opt for either the upper or lower layer for growth. The flow from (a) and the flow from (b), labeled with green-fluorescent dye, ran alongside each other without mixing in this environment (Fig 1A).

We established a condition where the upper layer contained minimal media with 1% glucose, while the lower layer lacked any carbon source. The growth of hyphae extending from spores trapped in (c) was observed for a duration of 18 h (Fig 1B and S1 Movie). Until 10 h, the number of hyphal tips in the upper and lower layers exhibited similarity, however after that, the number of tips increased in the upper layer while remaining constant in the lower layer (Fig 1C). The positions of hyphal tips are plotted over time 8 to 18 h (S1A Fig) and merged with different colors at different times (Fig 1D). The vertical distances from each tip and the boundary of the 2 layers was represented by a box plot (S1B Fig). At 400 μm from the trap exit, most hyphae extended in the upper layer (Fig 1D). The frequency of branching in the upper layer was 3 times more frequent than in the lower layer (*n* = 22 and 7). This observation implies that hyphal growth in the upper layer was more robust compared to the lower layer rather than chemotropism. Comparing growth on plates with glucose and without a carbon source, mycelium grows without a carbon source but has less branching and lower mycelial density than with glucose (S1C Fig). Aerial hyphae and conidiophore were hardly formed.

Subsequently, we examined the condition where both the upper and lower layers comprised a medium containing 1% glycerol, with 1% or 0.1% glucose exclusively introduced to the upper layer. Under this condition, there was notably more hyphal growth in the upper layer at 16 h (Figs 1E, S1D and S1E and S2 Movie). Tracking the hyphal tip positions reveals that the hyphae frequently retained their growth trajectory, remaining in the upper layer while transitioning from the upper layer to the lower layer (Fig 1F). The regulation of growth direction suggests a positive chemotropism toward glucose; however, the higher concentration of hyphae in the upper layer likely results from a combination of better growth in the upper layer and chemotropism towards glucose. As several overlapping hyphae with no visible tips were not accounted for by the number of hyphal tips, the area covered by hyphae was measured in both the upper and lower regions (S1F Fig).

For a quantitative assessment of various conditions, we tabulated the number of hyphal tips in both the upper and lower layers, calculating the percentage difference when the total approached approximately 40 to 60 (after 16 to 20 h). Beyond this point, an increase in the number of hyphae congests the flow paths, posing challenges for accurate response measurement. In the condition where both layers consisted of minimal medium containing glucose, the percent difference of the hyphal tip number between the upper and lower layers was 5 ± 4 (*n* = 4) (Fig 1G). In cases where only the upper layer contained 1% glucose and the lower layer contained no carbon source (Fig 1B), the difference was 39 ± 8 (*n* = 4). In cases where both layers contain 1% glycerol and only the upper layer additionally contains 1% glucose (Fig 1E), the value was 40 ± 5 (*n* = 4). Conversely, when both layers contained 1% glycerol, and only the upper layer featured 0.1% glucose, hyphae grew to a similar extent in both the upper and lower layers, with values dropping to 15 ± 6 (*n* = 4) (Figs 1G and S1G). No significant differences in colony size or mycelial density were observed on these plates (S1C Fig).

### Chemotropism to nitrogen

**Fig 2:**
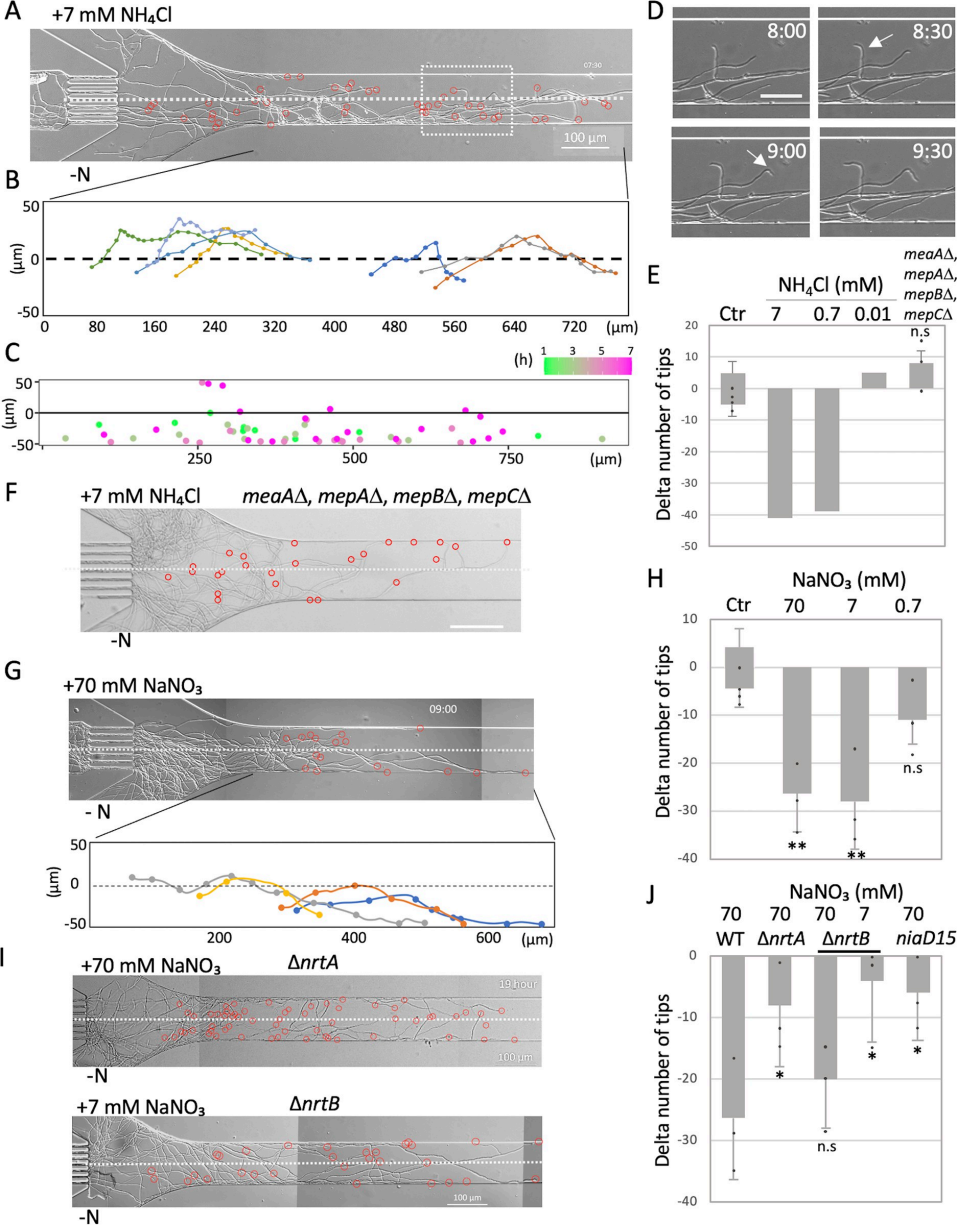
Chemotropism to nitrogen. (A) Hyphal growth in the condition with 7 mM NH4Cl (upper) and no nitrogen source (lower) from Movie 3. The positions of each hyphal tip are marked with red circles. The border of 2 layer is indicated by the dot line. Scale bar: 100 μm. (B) Tracking of directional change of hyphal tips around the border of 2 layers. Each trajectory is indicated by a different color. (C) Merged plots of the position of the hyphal tips in the channel at each time in different color over time. (D) Image sequence of hyphal growth direction change around 90 degrees around the border. The elapsed time is given in hours: minutes. Scale bar: 50 μm. (E) Difference in the percent of hyphal tips between the upper and lower space. The wild-type strain with and without 7, 0.7, or 0.07 mM NH4Cl in and the quadruple deletion strain of ammonium permease with and without 7 mM NH4Cl. Error bars (SD). *n* = 1 or 3; 40~50 hyphae in the experiments. ns, not significant. (F) Hyphal growth of meaA, mepA, mepB, mepC in the condition with 7 mM NH4Cl (upper) and no nitrogen source (lower). Scale bar: 100 μm. (G) Hyphal growth in the condition with 70 mM NaNO3 (upper) and no nitrogen source (lower) from S4 Movie. Scale bar: 100 μm. Tracking of directional change of hyphal tips around the border of 2 layers. Each trajectory is indicated by a different color. (H) Difference in the percent of hyphal tips between the upper and lower space. The wild-type strain with and without 70, 7, or 0.7 mM NaNO3. Error bars (SD). *n* = 3; 40~50 hyphae in 3 independent experiments. **, *p* ≤ 0.01; *, *p* ≤ 0.05; ns, not significant. (I) Hyphal growth in the condition with 70 or 7 mM NaNO3 (upper) and no nitrogen source (lower) in the ΔnrtA and ΔnrtB strains. The position of each hyphal tip is marked with a red circle. The border of 2 layers is indicated by the dot line. Scale bars: 100 μm. (J) Difference in the percent of hyphal tips to 70 mM NaNO3 in the wild type, ΔnrtA, ΔnrtB, and niaD15 mutant strains, and to 7 mM NaNO3 in the ΔnrtB strain. Error bars (SD). *n* = 3; 40~50 hyphae in 3 independent experiments. **, *p* ≤ 0.01; *, *p* ≤ 0.05; ns, not significant. The data underlying this figure can be found in S1 Data.

Nitrogen sources such as ammonia and nitrate are also important for mycelial growth. Mycelia grew on plates without a nitrogen source, but they were less branched and had lower mycelial density than plates with a nitrogen source, and few aerial mycelia and conidiophores formed (S2A Fig). We examined a condition in the device which the upper layer comprised minimal media with 7 mM ammonium chloride, while the lower layer lacked any nitrogen source. Despite no discernible difference in hyphal elongation rates between the upper and lower layers, numerous hyphae altered their growth direction from the upper layer back to the lower layer as they approached the upper layer (Figs 2A–2C and S2B and S3 Movie). Consequently, more hyphal tips extended into the lower layers (S2C Fig). Upon reaching the upper layer, certain hyphae underwent a change in direction of approximately 90 degrees (Fig 2D), which is not observed in the control where both layers contain 7 mM ammonium chloride. Since no significant difference in hyphal growth was observed in the case of 2 layers with and without chloride, the change of growth direction indicates a negative chemotropism toward ammonium.

Even when the ammonia concentration in the upper layer was diminished to 0.7 mM, a similar pattern persisted with more hyphae congregating in the lower layer (Fig 2E). The percent difference of the hyphal tip number between the upper and lower layers for 7 mM or 0.7 mM were approximately -40, respectively. However, when the ammonia concentration was further reduced to 10 μm, the uneven distribution of hyphae in the upper and lower layers ceased to be observed (Figs 2E and S2D). These findings indicate that the negative chemotropism to ammonia is concentration dependent.

*A*. *nidulans* possesses 4 ammonium permease genes, *meaA*, *mepA*, *mepB*, and *mepC* [21]. The strain with all 4 genes deleted exhibited growth defect on the minimal media with 7 mM ammonium chloride plate, similar to those without a nitrogen source (S2E Fig) [21]. Utilizing the microfluidic device, we examined the behavior of the quadruple deletion strain under conditions with and without 7 mM ammonium chloride. The negative chemotropism towards ammonia was no longer evident (Fig 2E and 2F), indicating the chemotropism is dependent on the ammonium permeases.

Similarly, chemotropism towards nitrate, an alternative nitrogen source, was examined using a medium with or without sodium nitrate. With a concentration of 70 mM sodium nitrate, more the hyphae grew in the lower layer, steering clear of the nitrate-containing medium (Figs 2G, 2H and S2F–S2H and S4 Movie); 7 mM sodium nitrate showed a similar degree of negative chemotropism (Fig 2H). With 0.7 mM sodium nitrate, the negative chemotropism was not significant (Figs 2H and S2I).

To examine the potential involvement of nitrate transporters in the negative chemotropism towards nitrate, we disrupted the genes, *nrtA* and *nrtB*, which encode nitrate transporters on the plasma membrane in *A*. *nidulans* [22]. It is shown that the transcript levels of *nrtA* and *nrtB* are enhanced in the presence of nitrate and repressed in the presence of ammonium. NrtA, characterized by a lower affinity for nitrate than NrtB, exhibits a faster transport rate, on the contrary, NrtB, with a higher affinity for nitrate than NrtA, enables to uptake nitrate even at low substrate concentrations [23]. Although the single deletion strain does not show significant growth defect on nitrate medium (S2E Fig), the double deletion is lethal under conditions with nitrate as the nitrogen source [23]. The Δ*nrtA* strain demonstrated a weakened negative chemotropism to 70 mM sodium nitrate (Fig 2I and 2J). In contrast, the Δ*nrtB* strain exhibited negative chemotropism to 70 mM sodium nitrate but diminished negative chemotropism to 7 mM sodium nitrate (Fig 2I and 2J). These results indicate that the negative chemotropism to nitrate is dependent on the nitrate transporters.

Since the nitrate reductase NiaD is essential for the growth of nitrate as a nitrogen source, the *niaD15* mutation results in a null nitrate reductase phenotype (S2E Fig) [23,24]. The *niaD15* mutant was cultivated in a medium containing ammonia, and the spores were subsequently harvested. Similarly, when the spores were placed under conditions with and without 70 mM sodium nitrate, the growth was notably delayed, and hyphae that did manage to grow to some extent did not exhibit any discernible chemotropism (Figs 2J and S2J).

### Chemotropism to low pH

**Fig 3:**
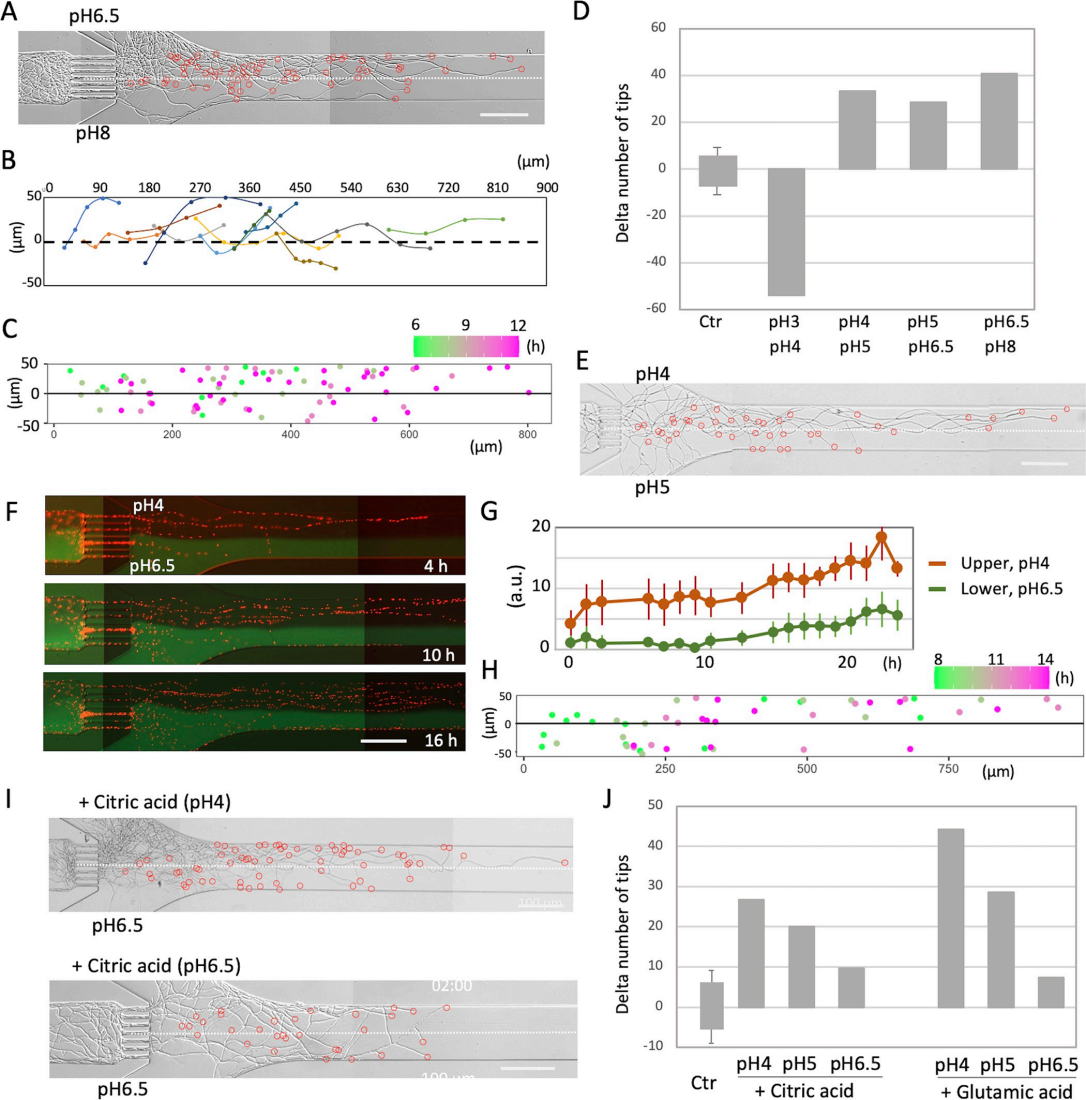
Chemotropism to pH. (A) Hyphal growth in the condition with pH 6.5 (upper) and pH 8 (lower) from S5 Movie. The positions of each hyphal tip are marked with red circles. The border of 2 layers is indicated by the dot line. Scale bars: 100 μm. (B) Tracking of directional change of hyphal tips around the border of 2 layers. Each trajectory is indicated by a different color. (C) Merged plots of the position of the hyphal tips in the channel at each time in different color over time. (D) Difference in the percent of hyphal tips in the combination of pH 3 and 4, pH 4 and 5, pH 5 and 6.5, and pH 6.5 and 8. *n* = 1; 40~50 hyphae in each experiment. (E) Hyphal growth in the condition with pH 4 (upper) and pH 5 (lower). The positions of each hyphal tip are marked with red circles. The border of 2 layers is indicated by the dot line. Scale bars: 100 μm. (F) Merged image sequence of hyphal growth of the strain expressing histone H1-mCherry and two-layers of pH 4 (upper) and pH 6.5 with green-fluorescent dye (lower) from Movie 6. The elapsed time is given in hours. Scale bar: 100 μm. (G) Time course of signal intensity of mCherry in arbitrary unit (a.u.) in the upper and lower layers. Error bars represent standard deviations (SD). *n* = 6 ROI (region of interest). (H) Merged plots of the position of the hyphal tips in the channel with the change in color over time from S6 Movie. (I) Hyphal growth in the condition with 3.4 mM citric acid pH 4 and no citric acid pH 6.5, and with 3.4 mM citric acid pH 6.5 and no citric acid pH 6.5. The positions of each hyphal tip are marked with red circles. The border of 2 layers is indicated by the dot line. Scale bars: 100 μm. (J) Difference in the percent of hyphal tips with/without 3.4 mM citric acid and 70 mM glutamic acid showing the results of 1 experiment with different pH 4, 5, and 6.5. The data underlying this figure can be found in S1 Data.

*A*. *nidulans* exhibits consistent growth across a broad pH range from 4 to 9 (S3A Fig). To assess chemotropism in response to different pH levels, the hyphae were cultivated in a medium with pH 6.5 for the upper layer and pH 8 for the lower layer. A higher number of hyphae selectively grew in the pH 6.5 layer (Figs 3A–3D and S3B and S5 Movie). Notably, many hyphae changed direction near the boundary, opting to remain in the pH 6.5 layer (Fig 3B). In the subsequent test with pH 5 and pH 6.5 media, more hyphae selectively grew in the pH 5 medium (Fig 3D). In the tests at pH 4 and pH 5, more hyphae selectively grew in the pH 4 medium (Fig 3D and 3E). In the pH 3 and pH 4 conditions, growth was inhibited in the pH 3 medium and some hyphae stopped growing, resulting in more mycelia growing in the pH 4 medium (Figs 3D and S3C). These outcomes indicate that the hyphae exhibit chemotropism towards pH 4 within the pH range of 3 to 8. In the chemotropism assays to this point using carbon and nitrogen sources (Figs 1 and 2), the pH of both the upper and lower layers was adjusted to eliminate any pH-related effects.

Chemotropism at pH 4 and pH 6.5 was assessed through fluorescence using the stain expresses histone H1-mCherry instead of counting tips of hyphae. Additionally, green-fluorescent dye was introduced into the pH 6.5 layer. The result indicated a higher preference for growth in the pH 4 medium, as more hyphae selectively thrived in the top layer (Figs 3F and S3D and S6 Movie). The number of hyphae in the upper and lower layers was quantified over time by evaluating fluorescence intensity (Fig 3F and 3G). The difference between the upper and lower layers is greater when hyphae volume is assessed by fluorescence intensity or area coverage in bright field than tracking the location and number of hyphae tips over time (Fig 3G and 3H).

We examined the tropism towards organic acids, specifically citric acid which serve as carbon sources and contribute to lowering the pH. The addition of 3.4 mM citric acid effectively decreased the pH of the minimal medium from 6.5 to 4. Under the condition where the upper layer comprised pH 4 medium containing 3.4 mM citric acid, and the lower layer consisted of pH 6.5 medium without citric acid, a greater number of hyphae selectively grew in the pH 4 citric acid medium (Fig 3I and 3J). Similar experiments were conducted using a medium containing 3.4 mM citric acid with adjusted pH values of 4 to 5 or 6.5. The chemotropism response was diminished at pH 5 and was not observed at pH 6.5 (Fig 3J). Parallel results were obtained with glutamic acid which serve as carbon and nitrogen sources and contribute to lowering the pH (Figs 3J and S3E). These findings suggest that the chemotropism of hyphae towards organic acids is influenced more by low pH than by nutrients.

### Role of proton pump and transcription factor PacC on pH chemotropism

**Fig 4:**
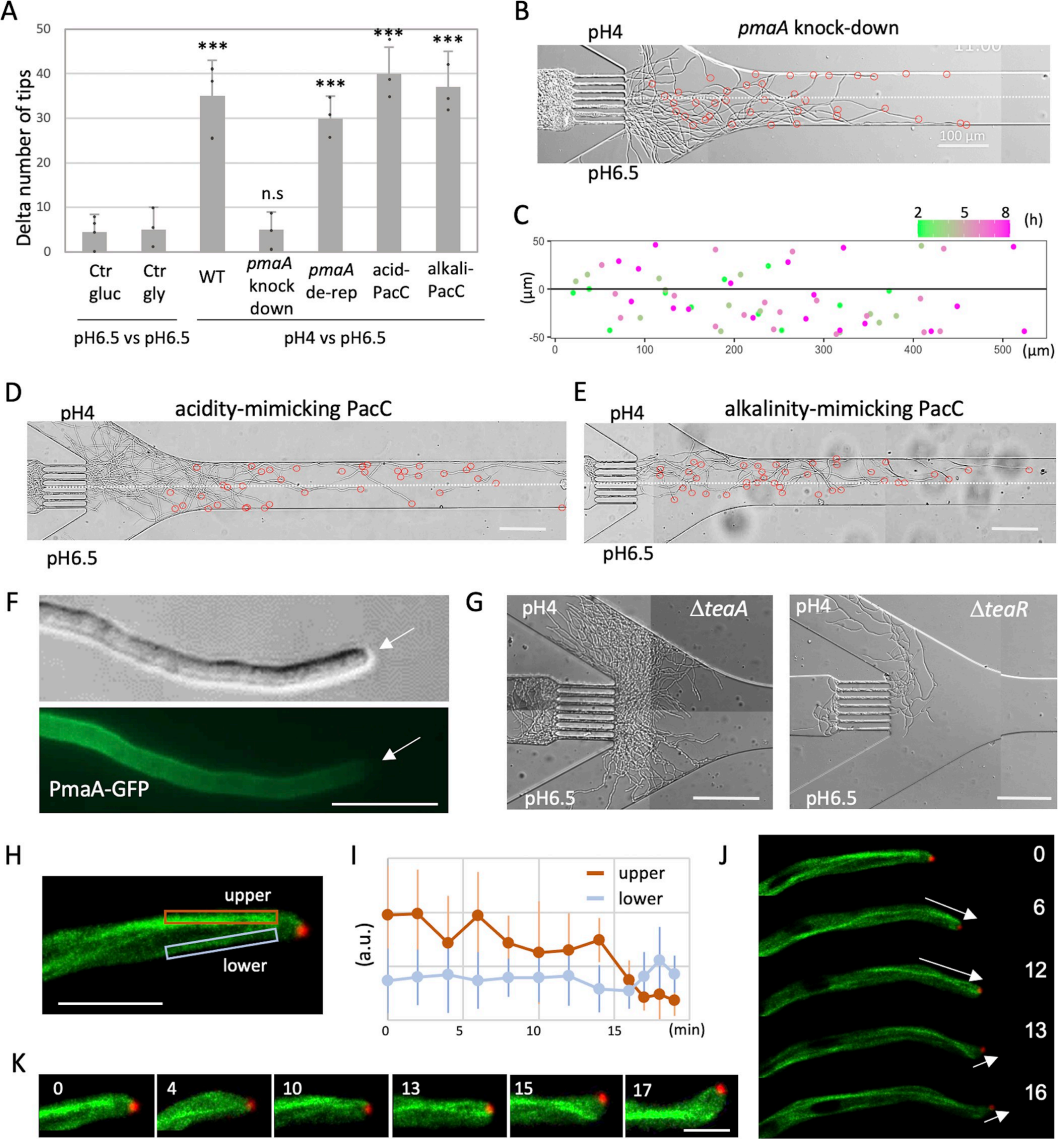
Molecular mechanism involved in chemotropism. (A) Difference in the percent of hyphal tips between pH 4 and pH6.5 in the wild type, pmaA knockdown strain (repressed in glucose, de-repressed in glycerol), acidity-mimicking and alkalinity-mimicking pacC mutants. The wild-type strain in identical 2 layers of pH 6.5 with glucose or pH 6.5 with glycerol as the control conditions. Error bars represent standard deviations (SDs). *n* = 3; 40~50 hyphae in 3 independent experiments. ***, *p* ≤ 0.001. (B) Hyphal growth in the condition with pH 4 (upper) and pH 6.5 (lower) in the pmaA knockdown strain repressed in glucose. The positions of each hyphal tip are marked with red circles. The border of 2 layers is indicated by the dot line. Scale bars: 100 μm. (C) Merged plots of the position of the hyphal tips in the channel with the change in color over time from (B). (D, E) Hyphal growth in the condition with pH 4 (upper) and pH 6.5 (lower) in the acidity-mimicking pacC mutant (D) and in the alkalinity-mimicking pacC mutant (E). The positions of each hyphal tip are marked with red circles. The border of 2 layer is indicated by the dot line. Scale bars: 100 μm. (F) DIC and GFP images of PmaA-GFP on the plasma membrane except the hyphal apex. The arrows indicate the apex of the hypha. Scale bar: 10 μm. (G) Hyphal growth in the condition with pH 4 (upper) and pH 6.5 (lower) in the ΔteaA and ΔteaR strain. Scale bars: 100 μm. (H) Fluorescence image of GFP-labeled microtubules and calmodulin CaM-mRFP. Scale bar: 5 μm. (I) Time course of signal intensity of GFP-microtubules in arbitrary unit (a.u.) in the upper and lower region shown in (H). Error bars represent SDs. *n* = 6 ROI (region of interest). (J) Image sequence of change in the hyphal growth direction and fluorescence image of GFP-microtubules and CaM-mRFP. The elapsed time is given in minutes. The arrows indicate the growth direction of the hypha. Scale bar: 5 μm. (K) Enlarged images of GFP-microtubules and CaM-mRFP at the hyphal tip of (J). The elapsed time is given in minutes. Scale bar: 2 μm. The data underlying this figure can be found in S1 Data.

Hyphae of *A*. *nidulans* maintain intracellular pH by expelling protons outside the cell through the plasma membrane proton pump, PmaA [25]. Pma1, an ortholog of the proton pump, is an essential gene in the yeast [26]. To investigate the role of PmaA, we utilized a knockdown strain where the expression of the *pmaA* gene was suppressed through carbon catabolite repression of the *alcA* promoter. This strain exhibited severe growth defects on a glucose medium plate but normal growth on a plate with glycerol as the carbon source (S4A Fig). The spores were harvested from the glycerol plate and introduced into the device under conditions of pH 4 and 6.5 with a glucose medium. The *pmaA* knockdown strain exhibited slow growth and lacked the chemotropism response to pH 4 (Fig 4A–4C). In contrast, wild-type hyphae selectively grew in the pH 4 medium (Fig 4A). Wild-type hyphae in the pH 4 medium changed their growth direction at the boundary with the pH 6.5 medium, repeatedly returning to the pH 4 layer. However, *pmaA* knockdown hyphae did not exhibit a clear preference for the 2 layers (Fig 4B and 4C). In the *pmaA* de-repressed condition with glycerol as carbon source, the chemotropism response to pH 4 was restored (Fig 4A). These results suggest that the chemotropism response to acid pH is PmaA dependent, although the repression of *pmaA* renders the cell unable to tolerate acidic pH, making it difficult to distinguish between lack of chemotropism and growth defects. Repression of *pmaA* was also implicated in negative chemotropism to nitrate (S4B Fig). Since repression of *pmaA* affects plasma membrane integrity and MAPK signaling as well as growth inhibition [27], it may also exert indirect effects, such as altering the localization of nitrate transporters.

Numerous fungi exhibit growth across a broad pH spectrum and their gene expression is adapted to the prevailing environmental pH. In *A*. *nidulans*, the transcription factor PacC serves as an activator for genes expressed under alkaline conditions and a repressor for those expressed under acidic conditions [28–32]. Under acidic conditions, the full-length form of PacC predominates but, under neutral-to-alkaline conditions, PacC undergoes sequential proteolytic cleavages. The processed PacC leave the zinc-finger region intact and acts as a repressor for genes expressed in acidic conditions and as an activator for genes expressed in alkaline conditions. To explore the relevance of PacC on chemotropism response to pH, we employed acidity-mimicking and alkalinity-mimicking *pacC* mutants, respectively, under the conditions pH 4 and 6.5 [33]. The acidity-mimicking *pacC* mutant still exhibited selective growth in the pH 4 medium (Fig 4A and 4D). Similarly, the alkalinity-mimicking *pacC* mutant selectively grew in the pH 4 medium (Fig 4A and 4E). These outcomes suggest that PacC does not play a role in the chemotropism response to acidity. The acidity-mimicking *pacC* mutant indicated growth defect on the plate with pH 6.5 and 9, whereas the alkalinity-mimicking *pacC* mutant did not (S4C Fig).

### Chemotropism and tip growth

GFP-tagged PmaA was observed to localize on the plasma membrane throughout hyphae but not at the tip region (Fig 4F). This localization pattern aligns with the observed pattern in *Neurospora crassa* [34]. The comparison of signal intensity of PmaA-GFP did not show significant difference between pH 4 and 6.5 in the device. Even though various plasma membrane transporters and pumps, including PmaA, are distributed throughout the membrane except at hyphal tips [35], the directional control of hyphal elongation is expected to occur at the tip. We explored the mechanisms linking these 2 phenomena, tip growth and chemotropism.

Polarity markers are localized at the apex of hyphal tips and play a crucial role in controlling growth direction (S4D Fig) [36,37]. This significance is highlighted by the observation that polarity marker mutants, Δ*teaA* and Δ*teaR*, exhibit zigzag and curved hyphal growth instead of a straight pattern (S4E Fig) [38]. To analyze the chemotropism of these mutants towards pH 4 and 6.5, a similar approach was employed. Upon exiting the channel, the hyphae of Δ*teaA* and Δ*teaR* mutants immediately changed direction, elongating upstream, but did not follow a straight growth path or make a clear choice between the upper and lower layers (Fig 4G). Notably, the chemotropism of these mutants to pH 4 appeared to be more sensitive, possibly due to the more frequent changes in growth direction observed in their hyphae.

Microtubules that consistently reach the hyphal tip play a pivotal role in controlling the position of polarity markers, consequently influencing growth machinery such as F-actin and the Spitzenkörper (SPK) [8,39–41]. When hyphae undergo a change in growth direction, the position of polarity markers and SPK shifts at the hyphal apex prior to the alteration in growth direction [37,40]. The fluorescent intensity of microtubules was compared between the upper and lower side of the hyphae when a change in growth direction occurred (Fig 4H and 4I). When the hypha changed the direction downward (Fig 4J, 0 to 12 min), the fluorescent intensity of the microtubules on the upper side of the hypha was higher than on the lower side (Fig 4I), and the SPK relocated to the lower side of the hyphal apex (Fig 4K). Conversely, when the hypha changed its direction upward (Fig 4J, 13 to 16 min), the fluorescent intensity of microtubules at the lower side exceeded that on the upper side (Fig 4J, 13 to 16 min), and the SPK relocated to the upper side of the hyphal apex (Figs 4K and S4F).

## Discussion

Assessing fungal growth in diverse nutrient and pH conditions is typically done by examining colony size. However, for an accurate evaluation of hyphal chemotropism, it becomes imperative to assess it at the cellular level. To achieve this, we aimed to establish a system that could precisely evaluate chemotropism at the cell level of hyphae through microenvironment control using a microfluidic device. As anticipated, the hyphae were more concentrated in the layer with glucose compared to the layer without glucose (Fig 1). At the interface of the layers, a change in the direction of hyphal growth was observed, favoring the layer with glucose, indicative of chemotropism toward glucose. Nevertheless, an evident increase in hyphal growth rate and branching was noted in the layer with glucose, suggesting enhanced growth. This highlights the challenge of distinguishing between augmented growth and chemotropism, even when evaluated at the cellular level.

This microfluidic device is engineered to observe both the directional growth response to chemical stimuli (chemotropism) and the differential growth between 2 environments. When the effect of growth difference between the 2 environments is negligible, the transition of hyphae from one region to the other clearly indicates chemotropism. The fungal growth is enhanced in the presence of nitrogen sources (S2A Fig) and remains unchanged across a pH range 4 to 9 (S3A Fig). Therefore, the evaluation of chemotropism to avoid ammonia and nitrate (Fig 2), as well as chemotropism to acidic pH (Fig 3), can be conducted independently of their impact on growth. This study highlights that adaptive growth to the environment are different from chemotropism. Ammonia and nitrate are toxic to cells but necessary for growth. The observed negative chemotropism, indicating an avoidance response, implies that growth proceeds by adapting to the environment, ensuring proper metabolism and utilization of these substances. Rapid fluctuations in ammonia and nitrate concentrations may be perceived as stressful due to their cellular toxicity in the hyphae. It is suggested that chemotropism to ammonia and nitrate should be studied under conditions featuring a gentle gradient to gain a more comprehensive understanding.

The transcription factor PacC plays a central role in adaptation to acidic and alkaline environments [28–33]. While PacC is essential for adaptation to shifts in pH towards acidity or alkalinity, it is not necessary for chemotropism to acid pH (Fig 4A, 4D and 4E). This also indicates that adaptive growth to the environment is different from chemotropism. Chemotropism is expected to manifest relatively quickly, responding within a short time frame. In contrast, adaptive growth to the environment necessitates a more extended period, often involving gene expression regulation. The differing temporal requirements for these responses likely account for the observed differences. Additionally, the presence of ammonia and nitrate might necessitate gene expression adjustments to metabolize substances that are essential but toxic.

The proton pump PmaA plays a role in chemotrophism towards pH 4 (Fig 4A–4C). PmaA-GFP is localized to the plasma membrane throughout the hyphae, excluding the hyphal tips (Fig 4F). This localization pattern suggests that proton efflux may not occur around the hyphal tips, potentially resulting in a lower pH near the tip of the hyphae, which could be the underlying reason for exhibiting chemotropism towards low pH. However, the cytoplasmic pH, visualized by the pH-sensitive probe in *Aspergillus niger*, appeared uniform throughout the hyphae [42]. The control of growth direction in hyphae occurs at the hyphal tip through the regulation of the localization of growth machinery. How do signals from transporters, which localize to the plasma membrane throughout the hyphae except at the hyphal tips, transmit to the hyphal tip? Microtubules, traveling along the plasma membrane and reaching the hyphal tip, emerge as a potential candidate. This is supported by the correlation between the signal intensity of microtubules on both sides of hyphae around the tips and the localization of growth machinery and growth direction (Fig 4H–4K). The activity of transporters may influence the polymerization and depolymerization of microtubules, thereby determining the position of polarity markers and the growth direction of hyphae (S4F Fig).

The metabolic capacity of fungi for various nutrient sources has been the subject of numerous studies, including genomic analysis, biochemical analysis, and transcriptional regulation mechanisms of related enzymes [43,44]. Employing this experimental system facilitates the exploration of the relationship between metabolic capacity and chemotropism towards various nutrient sources. For example, the chemotropism of different fungi may differ for monosaccharides other than glucose, disaccharides such as sucrose, and polysaccharides such as starch and cellulose. While the metabolic capabilities differ among various fungi, comparing chemotropism towards different nutrient sources using different fungal species would provide important insights into the ecology of fungi involved in organic matter decomposition.

The chemotropism of fungal hyphae towards phosphates and minerals would be the next challenge. The network of fungal hyphae spreads throughout the soil and penetrates the microspaces in soil aggregates [45]. The chemotropism should be crucial for the absorption and recovery of moisture, phosphate, and minerals present in the gaps of soil aggregates, enhancing the active search ability of hyphae [46]. This ability is not limited to the ecological role of fungi but becomes even more significant in mutualistic and endophytic associations with plant roots [47]. Plants obtain phosphate and minerals that have been recovered from the gaps in soil aggregates and the extensive network of hyphae beyond the reach of roots from symbiotic fungi [48].

Chemotropism towards organic acids and low pH could promote hyphal growth towards root exudates and allow hyphae to establish symbiotic/endophytic relationships with roots. Indeed, arbuscular mycorrhizal fungus, *Glomus mosseae*, has been documented to exhibit chemotropism towards compounds released by the roots of host plants [49]. However, this behavior can also attract fungal pathogens towards the roots [50]. This observation aligns with recent findings indicating that *F*. *oxysporum* germtubes exhibit chemotropism towards acidic pH [27]. Our experimental system holds potential for contributing to the identification of compounds in plant root extracts responsible for inducing chemotropism responses in mycorrhizal or endophytic fungi, as well as fungal pathogens, along with facilitating quantitative analyses of chemotropism.

Chemotaxis of unicellular organisms such as bacteria and neutrophils has been studied in several papers using microfluidic devices to create gradients of compounds [51,52]. However, no paper has yet created such a gradient and analyzed the chemotropism of fungal hyphae, which is expected to appear in the future. As in this paper, chemotaxis of T cells and neutrophils in a limited gradient in a Y-shaped microfluidic device has been investigated [53,54]. Various types of microfluidic devices have been developed to study chemotaxis in different organisms. Selecting the most suitable device requires a thorough understanding of its characteristics and the specific phenomena of the target organism. Chemotropism can be more complex than chemotaxis as it necessitates the evaluation of both directional control and growth. Our microfluidic device addresses this complexity by demonstrating its impact on both growth directional control and glucose-induced growth. Additionally, our device reveals negative chemotropism to nitrogen sources and positive chemotropism to acidic pH, along with the involvement of various related genes. This allows us to distinguish between the relatively rapid response of chemotropism and the slower environmental adaptation mediated through gene expression regulation.

## Materials and methods

### Fungal strains and media

A list of *A*. *nidulans* strains used in this study can be found in S1 Table. Supplemented minimal medium for *A*. *nidulans* is given in S2 Table. When auxotrophic strains were cultured, uridine was added at a final concentration of 0.6 mg/ml, uracil at 0.55 mg/ml, and pyridoxine at 0.4 mg/ml. The pH of the medium was adjusted with HCl or KOH.

### Gene disruption and GFP tagging

The 5′ end and 3′ end 1-kb sequence of the target gene were amplified by PCR using genomic DNA as template. The DNA fragment of *Af-pyrG* was also amplified by PCR. The 3 resulting DNA fragments were combined by the Fusion-PCR method [55]. The DNA fragment for GFP tagging at the C terminus of PmaA was amplified by fusion-PCR with a GFP-*Af-pyrG* cassette. The resulting gene cassettes were transformed into the TN02A3 strain (55). A list of primers used in this study is given in S3 Table.

### Microfluidic device

The microfluidic devices were designed by AutoCAD soft. The photomask was constructed by using μPG501 (Heidelberg Instruments). An acrylic plate cut in the shape of the designed channel was covered with photoresist (SU-8 3005&3010) and exposed to UV light (maskless lithography system DL-1000; Nano System Solutions) to cure the photoresist. The PDMS precursor (184 Silicone Elastomer Base, SYLGARD) and curing agent (184 Silicone Elastomer Curing Agent, SYLGARD) in a 10:1 ratio in a plastic cup and mixed vigorously with a medicine spoon for about 5 min to allow air to enter the mixture. After curing at 60°C for 24 h, PDMS was cut from the substrate using a cutter, and holes were drilled in the culture medium inlet and outlet portions at a total of 6 locations using a 1 mm diameter biopsy trepan (KAI industry). PDMS was bonded to the cover glass by plasma treatment (CUTE, Femoto Science).

### Microscopy

Cells were observed using epifluorescent inverted microscopy, including the Axio Observer Z1 (Carl Zeiss) microscope equipped with a Plan-Apochromat 63× 1.4 oil, 40× or 20× lens objective, an AxioCam 506 monochrome camera, and a Colibri.2 LED light (Carl Zeiss). The temperature of the stage was kept at 30°C by a thermo-plate (TOKAI HIT). A confocal laser scanning microscope LSM880 (Carl Zeiss) equipped with a 63×/0.9 numerical aperture Plan-Apochromat objective and a 40×/0.75 numerical aperture IR Achroplan W water immersion objectives (Carl Zeiss) were used to acquire confocal microscopic images. Images were collected and analyzed by the Zen system (Carl Zeiss) and ImageJ software.

### Microfluidic device imaging

The target strain is grown on agar medium to form conidiospores. The surface of the spore-forming colony was poked with a sterile toothpick, and the spores were suspended in about 100 μl of liquid medium in a micro tube. The spore suspension was diluted and pipetted into the inlet at (c) in the PDMS device (Fig 1A). A total of 5 ml medium was placed into a 10-ml plastic syringe (SS-10ESZ, Terumo) and connected to a polyethylene tube (inner diameter 0.38 mm, outer diameter 1.09 mm; BD intramedic). The air in the syringe was pushed out, then the tube was infused into the inlet (c) of the device. About 10 spores were trapped on a structure in the direction of the inflow of the spore suspension. The number of spores flowing to the front of the microfluidic channel was adjusted by observing under the microscope. The inlet (c) was closed by PDMS cylinder. To inject different media from inlets (a) and (b) in the device (Fig 1A), set the needle in a 10-ml syringe, connect the needle to the tube, and add about 7 ml of media. The air in the syringe was pushed out, then the tube was infused into the inlet (a) and (b) of the device. The green-fluorescent dye ATTO488 (Funakoshi) was added to only one of the media to confirm the formation of the 2 layers. The medium was pumped from the syringe through the tubing into the device using a positive displacement syringe pump (YSP-101, YMC) at a rate of 30 μl per hour. The device was fixed and incubated on a thermos-plate set at 30°C (TOKAI HIT). By Axio Observer Z1 (Carl Zeiss) microscope with a 20× objective lens, 3 to 5 horizontal fields of view were taken by the tiling function of the motorized stage. Three z-stacks (interval 3 μm) were set, then the images were taken every hour. The solution coming out of the outlet (d) was set to be sucked up with a tissue.

### Imaging analysis

For a quantitative assessment of various conditions, we counted the number of hyphal tips in both the upper and lower layers when the total approached approximately 40 to 50 (after 16 to 20 h). The percentages of tips in both the upper and lower layers were calculated, then the difference was compared among the conditions. MTrackJ plugin for ImageJ was used to track the hyphae tips as they elongated, and the coordinates of the hyphae tips at each time point tracked by MTrackJ were used to plot the tip trajectory. Coverage area by hyphae was also measured by ImageJ. Each image was converted to 8-bit grayscale, and the rolling ball algorithm was applied to eliminate the negative effects of lens aberrations. The area occupied by hyphae was determined by binarization.

## Supporting information

S1 Data Numerical data for all figures. (XLSX)

S1 Table Strains used in this study. (PDF)

S2 Table Composition of Minimal medium. (PDF)

S3 Table Primers used in this study. (PDF)

S1 Fig (A) Plots of the position of the hyphal tips in the channel at each time. The upper layer contains 1% glucose and the lower layer contains no carbon source from S1 Movie. (B) Box plots of vertical distance between hyphal tips and the boundary of the 2 layers with 1% glucose (upper) and no carbon source (lower) at each time point. (C) Mycelial growth on minimal media without a carbon source, with 1% glucose, 1% glycerol, and 1% glucose + 1% glycerol, and 500 spores of TH122 (no auxotrophic control strain) were inoculated on the center of plates and incubated for 7 days at 30°C. The hyphae around the edge of the colony grown on each medium were imaged by a zoom microscope. Scale bars: 1 mm. (D) Box plots of vertical distance between hyphal tips and the boundary of the 2 layers with 1% glycerol + 1% glucose (upper) and 1% glycerol (lower) at each time point. (E) Merged plots of the position of the hyphal tips in the channel of the 2 layers with 1% glycerol + 1% glucose (upper) and 1% glycerol (lower) at each time in different color over time. (F) Time course of area coverage by hyphae in 2 layer of 1% glycerol + 1% glucose (upper) and 1% glycerol (lower). (G) Hyphal growth in the condition with the upper layer containing 1% glycerol and 0.1% glucose and the lower layer containing 1% glycerol. The positions of each hyphal tip are marked with red circles. The border of 2 layer is indicated by the dot line. Scale bar: 100 μm. The data underlying this figure can be found in S1 Data.
(TIFF)

S2 Fig (A) Mycelial growth on minimal media without a nitrogen source, with 7, 0.7, 0.07 mM NH_4_Cl or 70, 7, 0.7 mM NaNO_3_, and 500 spores of TH122 (no auxotrophic control strain) were inoculated on the center of plates and incubated for 7 days at 30°C. The hyphae around the edge of the colony grown on each medium were imaged by a zoom microscope. Scale bars: 1 mm. (B) Box plots of vertical distance between hyphal tips and the boundary of the 2 layers with 7 mM NH_4_Cl (upper) and no nitrogen source (lower) at each time point from S3 Movie. (C) Time course of area coverage by hyphae in 7 mM NH_4_Cl (upper) and no nitrogen source (lower). (D) Hyphal growth in the condition with the upper layer containing 10 mM NH_4_Cl and the lower layer without a nitrogen source. The positions of each hyphal tip are marked with red circles. The border of 2 layers is indicated by the dot line. Scale bar: 100 μm. (E) Mycelial growth on minimal media without a nitrogen source, with 7 mM NH_4_Cl or 70 mM NaNO_3_, and 100 spores of TH122 (no auxotrophic control strain), the quadruple deletion strain of ammonium permease, *nrtA*D, *nrtB*D, *niaD15* mutant were inoculated on the center of plates and incubated for 5 days at 30°C. (F) Box plots of vertical distance between hyphal tips and the boundary of the 2 layers with 70 mM NaNO_3_ (upper) and no nitrogen source (lower) at each time point from S4 Movie. (G) Merged plots of the position of the hyphal tips in the channel with 70 mM NaNO_3_ (upper) and no nitrogen source (lower) at each time in different color over time. (H) Time course of area coverage by hyphae in 70 mM NaNO_3_ (upper) and no nitrogen source (lower). (I, J) Hyphal growth in the condition with the upper layer containing 0.7 mM NaNO_3_ and the lower layer without a nitrogen source (I). Hyphal growth of *niaD15* mutant in the condition with the upper layer containing 70 mM NaNO_3_ and the lower layer without a nitrogen source (J). The positions of each hyphal tip are marked with red circles. The border of 2 layers is indicated by the dot line. Scale bars: 100 μm. The data underlying this figure can be found in S1 Data.
(TIFF)

S3 Fig (A) Mycelial growth on minimal media with pH 4, 5, 6.5, 8, and 9. The 500 spores of TH122 (no auxotrophic control strain) were inoculated on the center of plates and incubated for 7 days at 30°C. (B) Time course of area coverage by hyphae in pH 6.5 (upper) and pH 8 (lower). (C) Hyphal growth in the condition with pH 3 (upper) and pH 4 (lower). The position of each hyphal tip is marked with a red circle. The border of 2 layers is indicated by the dot line. Scale bars: 100 μm. (D) Merged image sequence of hyphal growth (bright field) and two-layers of pH 4 (upper) and pH 6.5 with green-fluorescent dye (lower). The elapsed time is given in hours. Scale bar: 100 μm. (E) Hyphal growth in the condition with 70 mM glutamic acid pH 4 and no glutamic acid pH 6.5 (upper), and with 70 mM glutamic acid pH 6.5 and no glutamic acid pH 6.5 (lower). The position of each hyphal tip is marked with a red circle. The border of 2 layers is indicated by the dot line. Scale bars: 100 μm.
(TIFF)

S4 Fig (A) Mycelial growth of wild type and *alcA*(p)-*pmaA* strain on minimal media with 1% glucose or 1% glycerol; 100 spores of TN02A3 and SNT128 were inoculated on the center of plates and incubated for 3 days at 30°C. (B) Hyphal growth in the condition with NaNO_3_ (upper) and no nitrogen source (lower) in the *pmaA* knockdown strain repressed in glucose. The positions of each hyphal tip are marked with red circles. The border of 2 layers is indicated by the dot line. Scale bars: 100 μm. Difference in the percent of hyphal tips between with and without 7 mM NaNO_3_ in the wild type and *pmaA* knoc-kdown strain (repressed in glucose, de-repressed in glycerol). (C) Mycelial growth on minimal media with pH 4, 6.5, and 9. The 100 spores of TH122 (no auxotrophic control strain), acidity-mimicking *pacC* mutant, and alkalinity-mimicking *pacC* mutant were inoculated on the center of plates and incubated for 5 days at 30°C. (D) Localization of mRFP1-TeaA and GFP-TeaR at the hyphal apex. Scale bar: 2 μm. (E) The zigzag and curving hyphae of the Δ*teaA* and Δ*teaR* strain. Scale bars: 20 mm. (F) Putative link between chemotropism and tip growth. Hypothesis that differences in the activity of transporters on both sides of the plasma membrane transmit signals to the hyphae tip via microtubule activity.
(TIFF)

S1 Movie Hyphal growth in the condition with 1% glucose (upper) and no carbon source (lower). Every 2 h, total 18 h. Scale bar: 100 μm.
(AVI)

S2 Movie Hyphal growth in the condition with 1% glycerol + 1% glucose (upper) and 1% glycerol (lower). Every hour, total 16 h. Scale bar: 100 μm.
(AVI)

S3 Movie Hyphal growth in the condition with 7 mM NH_4_Cl (upper) and no nitrogen source (lower). Every 30 min, total 16 h. Without and with tracking. Scale bar: 100 μm.
(AVI)

S4 Movie Hyphal growth in the condition with 70 mM NaNO_3_ (upper) and no nitrogen source (lower). Every hour, total 9 h. Scale bar: 100 μm.
(AVI)

S5 Movie Hyphal growth in the condition with pH 6.5 (upper) and pH 8 (lower). Every hour, total 16 h. Without and with tracking. Scale bar: 100 μm.
(AVI)

S6 Movie Hyphal growth in the condition pH 4 (upper) and pH 6.5 with green-fluorescent dye (lower). DIC and green merged images, every hour in total 17 h. Green and red merged images, every hour in total 22 h. Scale bar: 100 μm.
(AVI)
