# Supplementary material for: Hyphae of the fungus Aspergillus nidulans demonstrate chemotropism to nutrients and pH
Source: PLoS Biol. 2024 Jul 30;22(7):e3002726. doi: 10.1371/journal.pbio.3002726 (PMC11288418; doi:10.1371/journal.pbio.3002726)
Supplement: S1 Table — (PDF) [file pbio.3002726.s002.pdf]

Table S1 Strains used in this study

Strain	Genotype	Source
A.nidulans TN02A3	*pyrG89, argB2, ΔnkuA::argB, pyroA4*	1
TH122	*pyrG89, argB2, ΔnkuA::argB, pyroA4, riboB2, riboB2::riboB*、*HH1-mCherry::AfpyrG*、 *wA::Lifeact-GFP::AfpyroA*	This study
RH1 (*ΔnrtA*)	*pyrG89, argB2, ΔnkuA::argB, pyroA4, ΔnrtA(AN1008)::pyrG89*	This study
RH2 (*ΔnrtB*)	*pyrG89, argB2, ΔnkuA::argB, pyroA4, ΔnrtB (AN0399)::pyrG89*	This study
SNT128	pNT65 in TN02A3, *pmaA* is replaced with *alcA(p)-pmaA-mEosFP::pyrG, pyroA4*	This study
RH6	*pyrG89, argB2, ΔnkuA::argB, pmaA-GFP::pyrG*	This study
MH11226	*biA1, amdS-lacZ, pyroA4, meaAΔ::argB, mepAΔ::riboB, mepBΔ::Ble^R, mepCΔ::Bar veA1*	2
RT108 (A691)	*biA1, niaD15, veA1*	3
MAD1081	*yA2, adE20, pacCc202, pacC+/-20205* (acidity-mimicking PacC)	4
MAD306	*pabaA1, pacCc202* (alkalinity-mimicking PacC)	4
SNT30	*pyrG89, ΔargB::trpCΔB, ΔteaA::argB*	5
SNT33	*pyrG89, ΔargB::trpCΔB, pyroA4, ΔteaR::pyrG*	5

References

1. T. Nayak, E. Szewczyk, C. E. Oakley, A. Osmani, L. Ukil, S. L. Murray, M. J. Hynes, S. A. Osmani, B. R. Oakley. A versatile and efficient gene-targeting system for *Aspergillus nidulans*. *Genetics,* **172**, 1557-66 (2006).
2. B. J. Monahan, M. C. Askin, M. J. Hynes, M. A. Davis. Differential expression of *Aspergillus nidulans* ammonium permease genes is regulated by GATA transcription factor AreA. *Eukaryot. Cell*., **5**, 226-237 (2006).
3. D. J. Cove. Genetic studies of nitrate assimilation in *Aspergillus nidulans*. *Biol. Rev.* **54**, 291-327 (1979).
4. E. A. Espeso, T. Roncal, E. Diez, L. Rainbow, E. Bignell, J. Alvaro, T. Suarez, S. H. Denison, J. Tilburn, H. N. Arst Jr., M. A. Peñalva. On how a transcription factor can avoid its proteolytic activation in the absence of signal transduction. *EMBO J*. **19**, 719-728 (2000).
5. N. Takeshita, Y. Higashitsuji, S. Konzack, R. Fischer. Apical sterol-rich membranes are essential for localizing cell end markers that determine growth directionality in the filamentous fungus *Aspergillus nidulans*. *Mol. Biol. Cell*,**19**, 339-51 (2008).
