# Supplementary material for: Hyphae of the fungus Aspergillus nidulans demonstrate chemotropism to nutrients and pH
Source: PLoS Biol. 2024 Jul 30;22(7):e3002726. doi: 10.1371/journal.pbio.3002726 (PMC11288418; doi:10.1371/journal.pbio.3002726)
Supplement: S2 Table — (PDF) [file pbio.3002726.s003.pdf]

Table S2. Composition of Minimal medium

Minimal Medium	
Glucose	10 g
$NaNO_3$	6 g
KH_2PO4	1.52 g
KCl	0.52 g
$MgSO_4 \cdot 7H_2O$	0.52 g
Hunter's Trace element	2 mL
pH	6.5
per litter	

Hunter's Trace element	
$ZnSO_4 \cdot 7H_2O$	2.2 g
H_3BO_3	1.1 g
$MnCl_2 \cdot 4H_2O$	0.5 g
$FeSO_4 \cdot 7H_2O$	0.5 g
$CoCl_2 \cdot 6H_2O$	0.16 g
$CuSO_4 \cdot 5H_2O$	0.16 g
$(NH_4)_6Mo_7O_{24} \cdot 4H_2O$	0.11 g
per 100 mL	
