# Supplementary material for: Hyphae of the fungus Aspergillus nidulans demonstrate chemotropism to nutrients and pH
Source: PLoS Biol. 2024 Jul 30;22(7):e3002726. doi: 10.1371/journal.pbio.3002726 (PMC11288418; doi:10.1371/journal.pbio.3002726)
Supplement: S3 Table — (PDF) [file pbio.3002726.s004.pdf]

Table S3. Primers used in this study

Primer	Sequence (5'-3')
pyrG fw	GCCTCAAACAATGCTCTTCA
pyrG rv	CTGTCTGAGAGGAGGCACTG
nrtA up fw	GAGCCGTCAGCACATACTT
nrtA up rv	CCAGCGCCTGCACCAGCTCCCTCCCTCATCTGACTTTTCG
nrtA dw fw	CAGTGCCTCCTCTCAGACAGTAGTTGACCGTGTGATTGGG
nrtA dw rv	CTCGATACTGCTGCTGAAGG
nrtA up fw nest	GGAGATCGTGGACATTTTTG
nrtA dw rv nest	AACATTGCGAGACTAAACGC
nrtB up fw	AAGAACATTGTTGGTACTGCC??
nrtB up rv	GGTGAAGAGCATTGTTTGAGGCTGCTTCAGATATTCGTGG
nrtB dw fw	GCCTCCTCTCAGACAGTAGACGCCATCTTCAAGTTTCGCG
nrtB dw rv	ATGTATTCCATTGAGGCAGG
nrtB up fw nest	CGGCTATCTACGACTCCTTC
nrtB dw rv nest	CATGCAACCCACCTAGTATG
pmaA up fw	GTCGTCGAGATGCTTCAG
pmaA up rv	CCAGCGCCTGCACCAGCTCCCTCATCGTCCTCAGGCTC
pmaA dw fw	CAGTGCCTCCTCTCAGACAGGGATATCACTTGCTCATACATG
pmaA dw rv	GCAGAATCTATGACTACGACG
ga5 fw	GGAGCTGGTGCAGGCGCTGG
pmaA up fw nest	GTCACTTGACTGCCATGAC
pmaA dw rv nest	CTATGACTACGACGAACGC
