# Supplementary figures and images for: Hyphae of the fungus Aspergillus nidulans demonstrate chemotropism to nutrients and pH
Source: PLoS Biol. 2024 Jul 30;22(7):e3002726. doi: 10.1371/journal.pbio.3002726 (PMC11288418; doi:10.1371/journal.pbio.3002726)

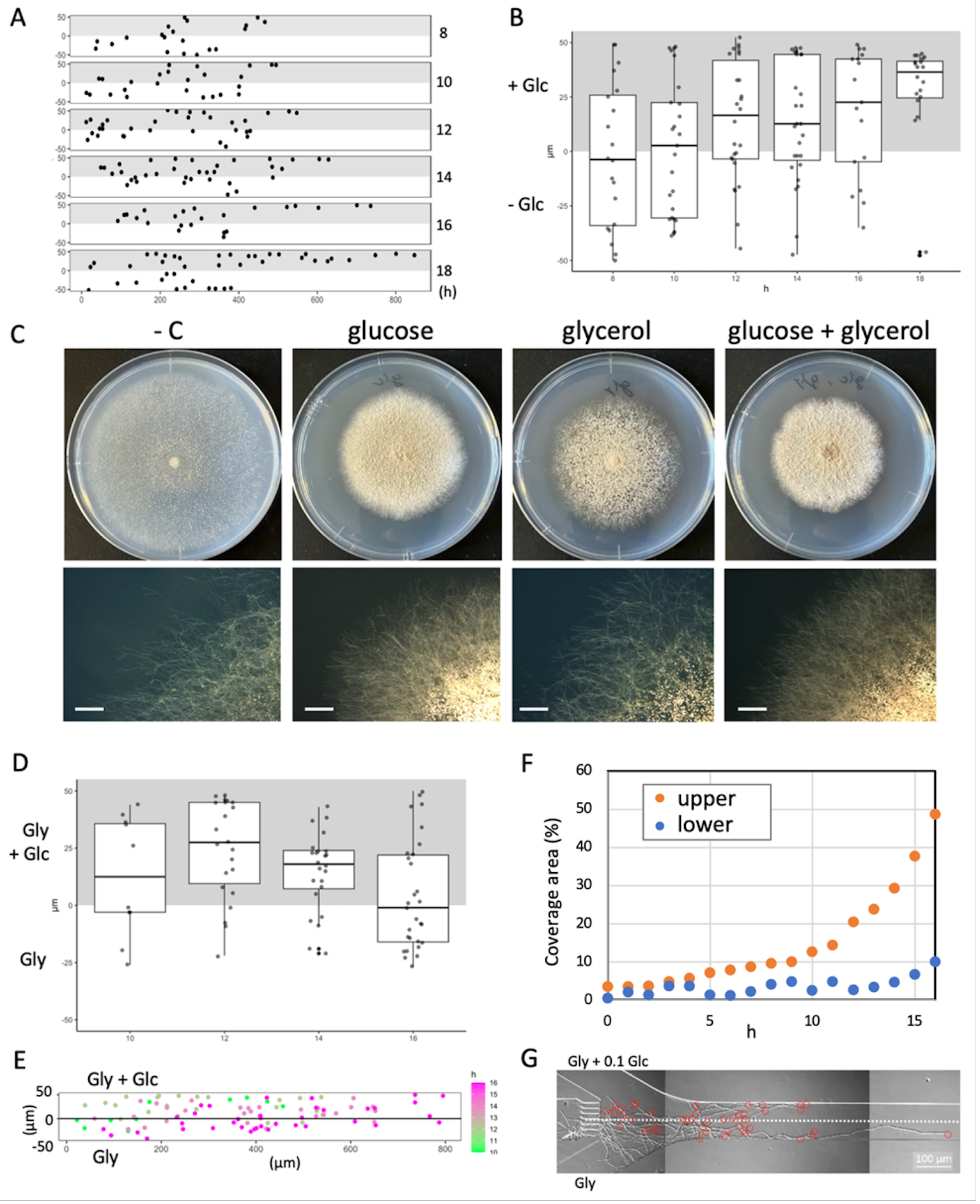

Supplement: S1 Fig — (A) Plots of the position of the hyphal tips in the channel at each time. The upper layer contains 1% glucose and the lower layer contains no carbon source from S1 Movie. (B) Box plots of vertical distance between hyphal tips and the boundary of the 2 layers with 1% glucose (upper) and no carbon source (lower) at each time point. (C) Mycelial growth on minimal media without a carbon source, with 1% glucose, 1% glycerol, and 1% glucose + 1% glycerol, and 500 spores of TH122 (no auxotrophic control strain) were inoculated on the center of plates and incubated for 7 days at 30°C. The hyphae around the edge of the colony grown on each medium were imaged by a zoom microscope. Scale bars: 1 mm. (D) Box plots of vertical distance between hyphal tips and the boundary of the 2 layers with 1% glycerol + 1% glucose (upper) and 1% glycerol (lower) at each time point. (E) Merged plots of the position of the hyphal tips in the channel of the 2 layers with 1% glycerol + 1% glucose (upper) and 1% glycerol (lower) at each time in different color over time. (F) Time course of area coverage by hyphae in 2 layer of 1% glycerol + 1% glucose (upper) and 1% glycerol (lower). (G) Hyphal growth in the condition with the upper layer containing 1% glycerol and 0.1% glucose and the lower layer containing 1% glycerol. The positions of each hyphal tip are marked with red circles. The border of 2 layer is indicated by the dot line. Scale bar: 100 μm. The data underlying this figure can be found in S1 Data. (TIFF) [file pbio.3002726.s005.tiff]

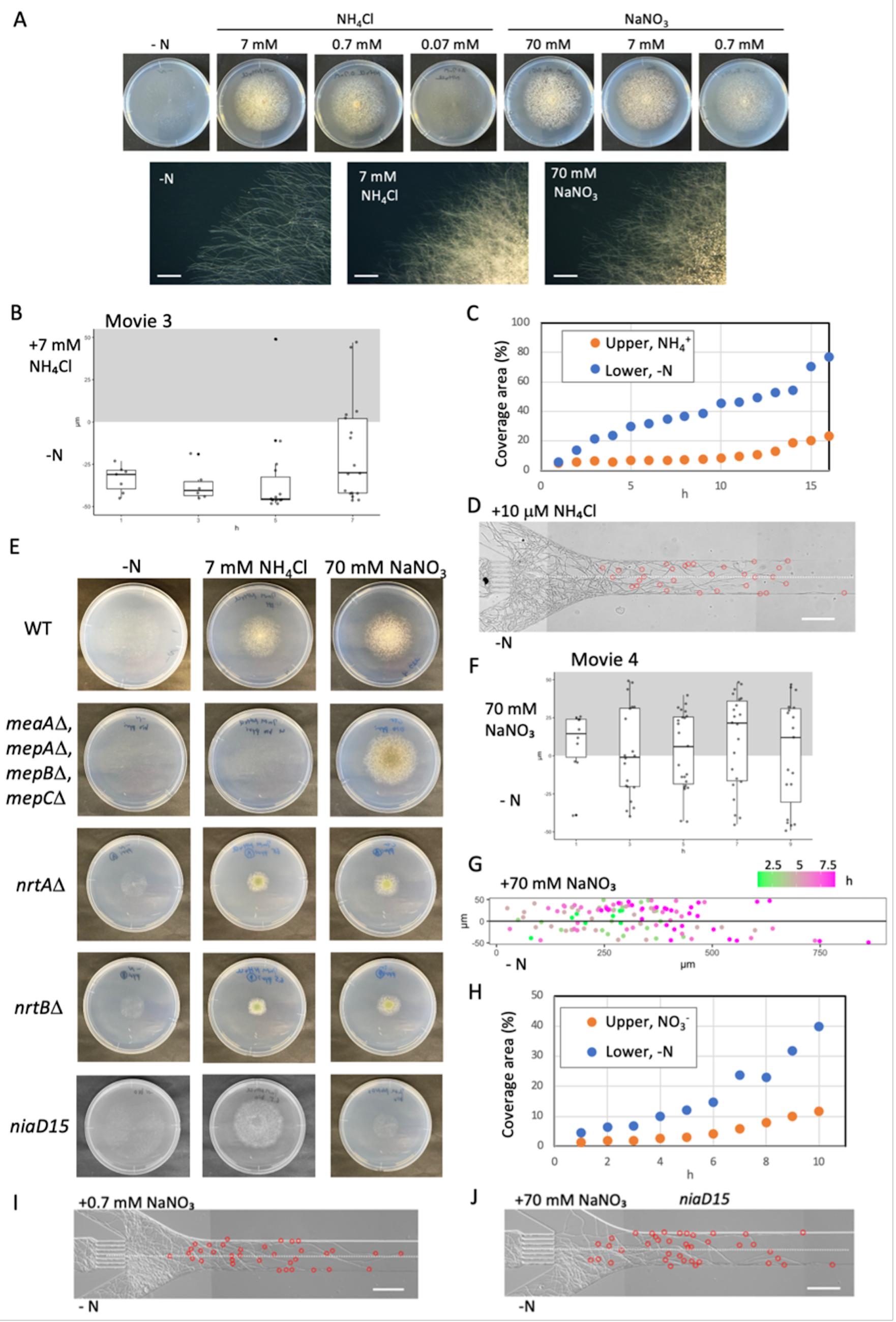

Supplement: S2 Fig — (A) Mycelial growth on minimal media without a nitrogen source, with 7, 0.7, 0.07 mM NH4Cl or 70, 7, 0.7 mM NaNO3, and 500 spores of TH122 (no auxotrophic control strain) were inoculated on the center of plates and incubated for 7 days at 30°C. The hyphae around the edge of the colony grown on each medium were imaged by a zoom microscope. Scale bars: 1 mm. (B) Box plots of vertical distance between hyphal tips and the boundary of the 2 layers with 7 mM NH4Cl (upper) and no nitrogen source (lower) at each time point from S3 Movie. (C) Time course of area coverage by hyphae in 7 mM NH4Cl (upper) and no nitrogen source (lower). (D) Hyphal growth in the condition with the upper layer containing 10 mM NH4Cl and the lower layer without a nitrogen source. The positions of each hyphal tip are marked with red circles. The border of 2 layers is indicated by the dot line. Scale bar: 100 μm. (E) Mycelial growth on minimal media without a nitrogen source, with 7 mM NH4Cl or 70 mM NaNO3, and 100 spores of TH122 (no auxotrophic control strain), the quadruple deletion strain of ammonium permease, nrtAD, nrtBD, niaD15 mutant were inoculated on the center of plates and incubated for 5 days at 30°C. (F) Box plots of vertical distance between hyphal tips and the boundary of the 2 layers with 70 mM NaNO3 (upper) and no nitrogen source (lower) at each time point from S4 Movie. (G) Merged plots of the position of the hyphal tips in the channel with 70 mM NaNO3 (upper) and no nitrogen source (lower) at each time in different color over time. (H) Time course of area coverage by hyphae in 70 mM NaNO3 (upper) and no nitrogen source (lower). (I, J) Hyphal growth in the condition with the upper layer containing 0.7 mM NaNO3 and the lower layer without a nitrogen source (I). Hyphal growth of niaD15 mutant in the condition with the upper layer containing 70 mM NaNO3 and the lower layer without a nitrogen source (J). The positions of each hyphal tip are marked with red circles. The border of 2 lay [file pbio.3002726.s006.tiff]

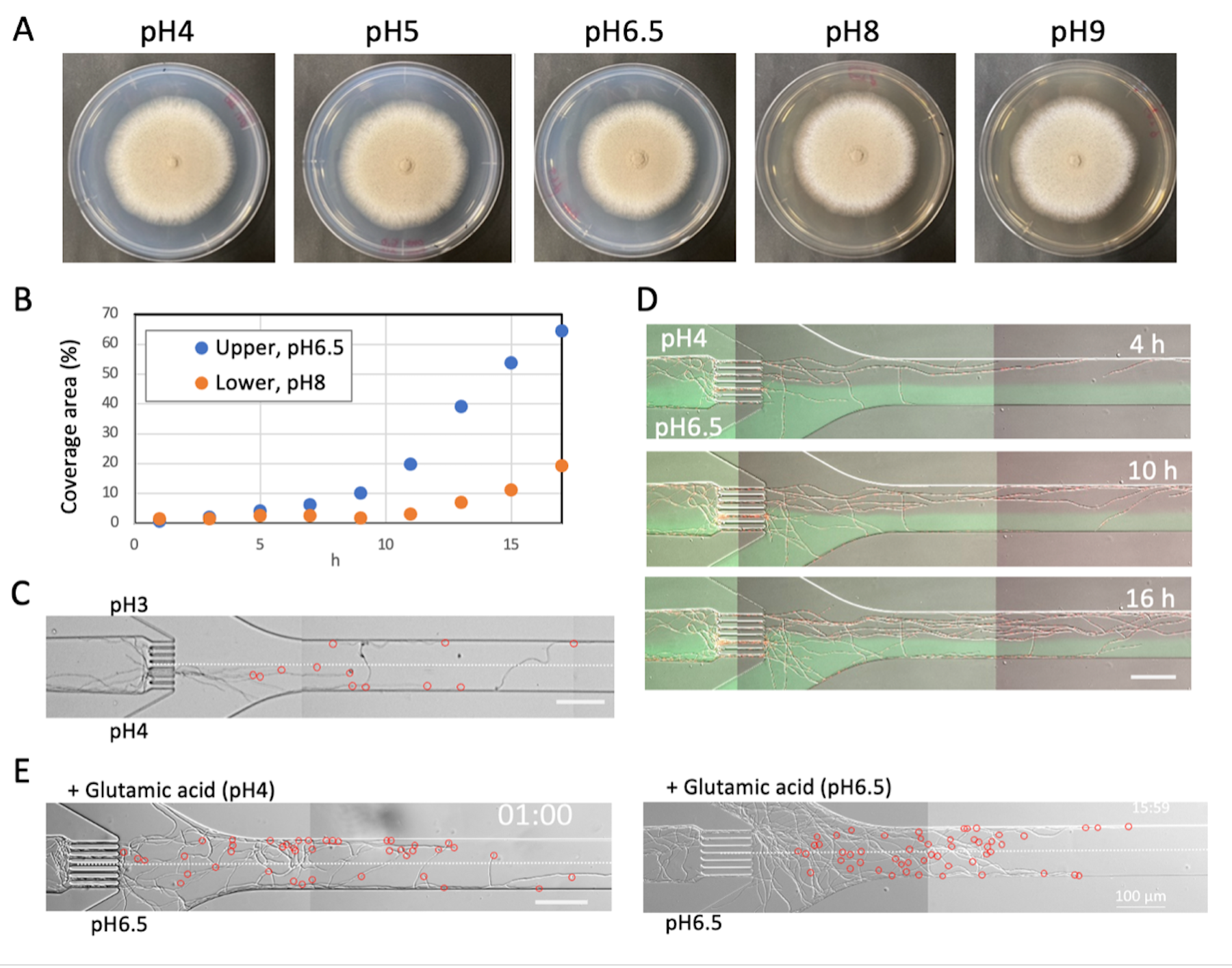

Supplement: S3 Fig — (A) Mycelial growth on minimal media with pH 4, 5, 6.5, 8, and 9. The 500 spores of TH122 (no auxotrophic control strain) were inoculated on the center of plates and incubated for 7 days at 30°C. (B) Time course of area coverage by hyphae in pH 6.5 (upper) and pH 8 (lower). (C) Hyphal growth in the condition with pH 3 (upper) and pH 4 (lower). The position of each hyphal tip is marked with a red circle. The border of 2 layers is indicated by the dot line. Scale bars: 100 μm. (D) Merged image sequence of hyphal growth (bright field) and two-layers of pH 4 (upper) and pH 6.5 with green-fluorescent dye (lower). The elapsed time is given in hours. Scale bar: 100 μm. (E) Hyphal growth in the condition with 70 mM glutamic acid pH 4 and no glutamic acid pH 6.5 (upper), and with 70 mM glutamic acid pH 6.5 and no glutamic acid pH 6.5 (lower). The position of each hyphal tip is marked with a red circle. The border of 2 layers is indicated by the dot line. Scale bars: 100 μm. (TIFF) [file pbio.3002726.s007.tiff]

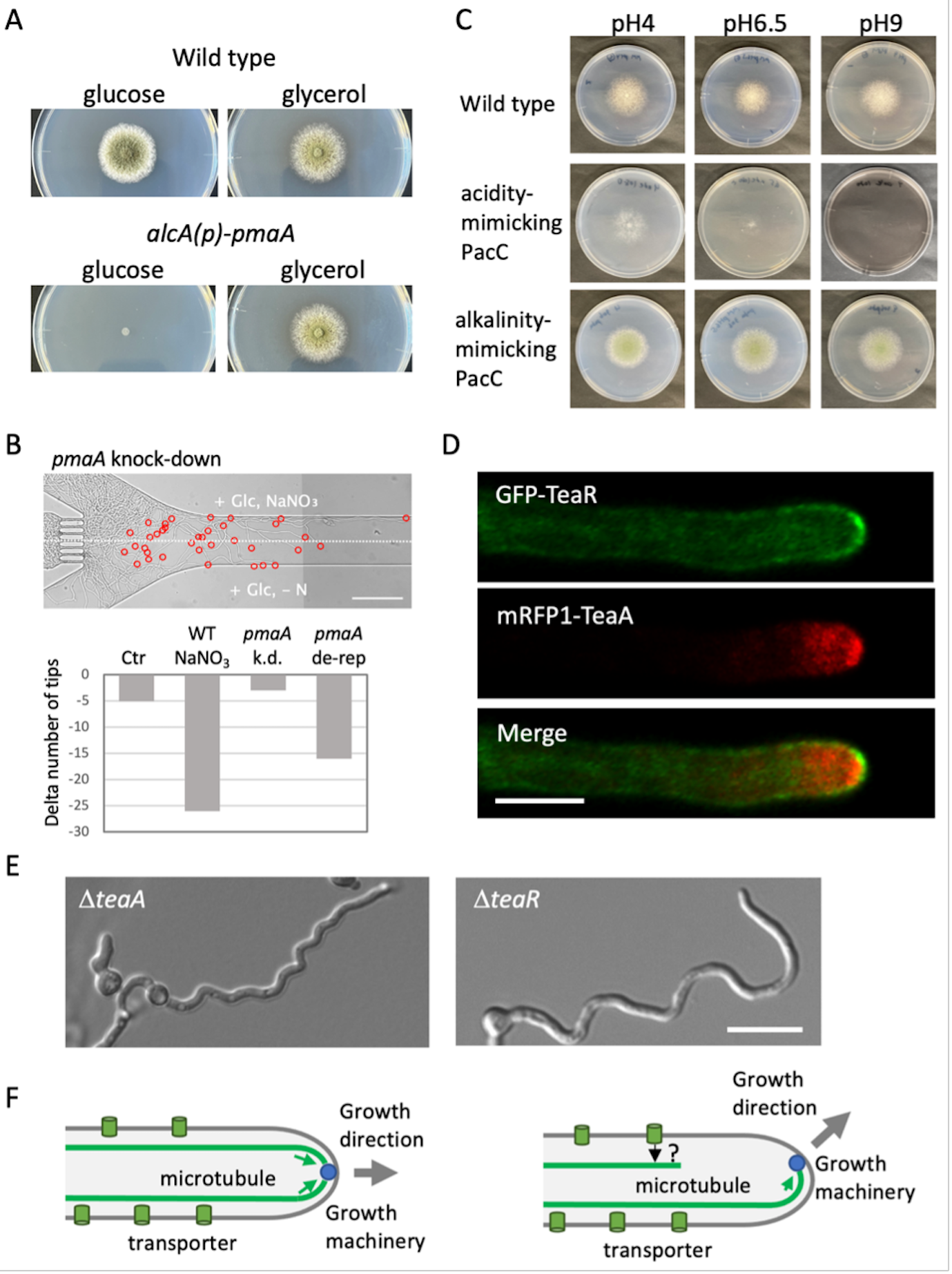

Supplement: S4 Fig — (A) Mycelial growth of wild type and alcA(p)-pmaA strain on minimal media with 1% glucose or 1% glycerol; 100 spores of TN02A3 and SNT128 were inoculated on the center of plates and incubated for 3 days at 30°C. (B) Hyphal growth in the condition with NaNO3 (upper) and no nitrogen source (lower) in the pmaA knockdown strain repressed in glucose. The positions of each hyphal tip are marked with red circles. The border of 2 layers is indicated by the dot line. Scale bars: 100 μm. Difference in the percent of hyphal tips between with and without 7 mM NaNO3 in the wild type and pmaA knoc-kdown strain (repressed in glucose, de-repressed in glycerol). (C) Mycelial growth on minimal media with pH 4, 6.5, and 9. The 100 spores of TH122 (no auxotrophic control strain), acidity-mimicking pacC mutant, and alkalinity-mimicking pacC mutant were inoculated on the center of plates and incubated for 5 days at 30°C. (D) Localization of mRFP1-TeaA and GFP-TeaR at the hyphal apex. Scale bar: 2 μm. (E) The zigzag and curving hyphae of the ΔteaA and ΔteaR strain. Scale bars: 20 mm. (F) Putative link between chemotropism and tip growth. Hypothesis that differences in the activity of transporters on both sides of the plasma membrane transmit signals to the hyphae tip via microtubule activity. (TIFF) [file pbio.3002726.s008.tiff]
